# Queen and young larval pheromones impact nursing and reproductive physiology of honey bee (*Apis mellifera*) workers

**DOI:** 10.1007/s00265-014-1811-y

**Published:** 2014-09-25

**Authors:** Kirsten S. Traynor, Yves Le Conte, Robert E. Page

**Affiliations:** 1 School of Life Sciences, Arizona State University, Tempe, AZ USA; 2 INRA, UR 406, Abeilles et Environnement, Site Agroparc, 84914 Avignon, France

**Keywords:** Honey bee, Brood pheromone, Queen mandibular pheromone, e-Beta ocimine, Hypopharyngeal gland, Reproductive ground plan

## Abstract

Several insect pheromones are multifunctional and have both releaser and primer effects. In honey bees (*Apis mellifera*), the queen mandibular pheromone (QMP) and e-beta-ocimene (eβ), emitted by young worker larvae, have such dual effects. There is increasing evidence that these multifunctional pheromones profoundly shape honey bee colony dynamics by influencing cooperative brood care, a fundamental aspect of eusocial insect behavior. Both QMP and eβ have been shown to affect worker physiology and behavior, but it has not yet been determined if these two key pheromones have interactive effects on hypopharyngeal gland (HPG) development, actively used in caring of larvae, and ovary activation, a component of worker reproductive physiology. Experimental results demonstrate that both QMP and eβ significantly suppress ovary activation compared to controls but that the larval pheromone is more effective than QMP. The underlying reproductive anatomy (total ovarioles) of workers influenced HPG development and ovary activation, so that worker bees with more ovarioles were less responsive to suppression of ovary activation by QMP. These bees were more likely to develop their HPG and have activated ovaries in the presence of eβ, providing additional links between nursing and reproductive physiology in support of the reproductive ground plan hypothesis.

**Electronic supplementary material:**

The online version of this article (doi:10.1007/s00265-014-1811-y) contains supplementary material, which is available to authorized users.

Within social insects, the chemical communication system has proven to be highly diversified and richly complex, enhanced by synergistic interactions and context-dependent messaging (Slessor et al. 2005). For example, at least 50 substances derived from queens, workers, and immatures are expressed within the colonies of honey bees (*Apis mellifera*) (reviewed in Pankiw 2004). A number of chemicals act as releasers of behavior (releaser pheromones), causing rapid but short-lived responses, such as the attraction/orienting behavior in response to pheromone emission from the dorsal Nasanov gland (Free 1987; Pickett et al. 1980). Other chemicals act as primers (primer pheromones) and slowly influence behavior through long-term physiological effects, thereby influencing broad aspects of colony organization, caste structure, and the division of labor (Le Conte and Hefetz 2008; Wilson and Bossert 1963; Winston and Slessor 1998). Several multifunctional pheromones have both releaser and primer effects, such as queen mandibular pheromone (QMP) and brood ester pheromones (BEPs) produced by larvae. There is increasing evidence that these multifunctional pheromones may have profound effects in shaping honey bee colony dynamics (reviewed in Alaux et al. 2010; Winston and Slessor 1998).

One of the primary effects elicited by honey bee pheromones is the organization of care received by immature bees. Larvae are confined to a cell, cannot feed themselves, and must signal their needs to adult nurses. By emitting pheromones, the larvae influence the behavior and physiology of their nurses, stimulating them to provide appropriate nutritional resources (Arnold et al. 1994; Le Conte et al. 2001; Maisonnasse et al. 2010; Mohammedi et al. 1996; Mohammedi et al. 1998; Pankiw et al. 1998; Sagili and Pankiw 2009). Pheromone composition changes as larvae age, with young larvae emitting the volatile pheromone e-beta ocimene (eβ) and old larvae predominantly emitting a blend of ethyl and methyl fatty acid esters known collectively as BEPs. Nurse bees tightly regulate larval growth by adjusting the larval feeding regime according to larval age (Leimar et al. 2012; Linksvayer et al. 2011; Wang et al. 2014), indicating that nurse bees may use larval pheromones to regulate larval diet and prime their physiology for brood care (Le Conte et al. 1994, 1995; Mohammedi et al. 1996).

Both queens and brood emit primer pheromones that strongly impact cooperative brood care, a redundancy in control mechanisms that appears to be a common feature of pheromone-based signaling systems in eusocial insects (Hoover et al. 2003). Queens produce QMP, known to release a worker retinue response and impact worker behavior through induced changes to their endocrine and reproductive physiology (De Groot and Voogd 1954; Jay 1970, 1972; Jay and Jay 1976; Kaatz et al. 1992). Both QMP and BEP of older larvae suppress ovary activation and stimulate hypopharyngeal gland (HPG) development of facultatively sterile workers, priming them to forego reproduction and activate both HPG and mandibular glands for brood care (Hoover et al. 2003; Mohammedi et al. 1996, 1998; Peters et al. 2010). The paired HPGs of nurse-aged bees produce the protein-rich food fed to developing larvae (Snodgrass 1925). To activate their HPG, bees normally must consume protein and have contact with larvae for 3 days (Huang et al. 1989; Huang and Otis 1989). Young adult bees receive proportionally more brood food from nurse-aged bees than older bees (Crailsheim 1991, 1992). This protein-rich diet can trigger activation of the HPGs in young workers while poor worker nutrition negatively impacts HPG development (Peters et al. 2010). A restricted diet also suppresses ovary activation, because bees do not have the protein resources to develop oocytes (Hoover et al. 2006; Lin and Winston 1998). Recent research has shown that simultaneous exposure to QMP and BEP even in the absence of a protein resource can increase protein production in HPGs (Peters et al. 2010) suggesting that under the queenright conditions of a hive environment (i.e., presence of queen and brood pheromones), workers can catabolize bodily proteins for larval food production. BEP stimulates increased pollen foraging, which is directly canalized into rearing more brood (Sagili and Pankiw 2009; Sagili et al. 2011).

The reproductive ground plan hypothesis proposes that reproductive physiology provided building blocks on which natural selection acted to establish a foraging division of labor. As originally proposed, bees with more ovarioles and higher titers of the egg yolk precursor vitellogenin bias their foraging toward pollen collection used for larval rearing, repurposing reproductive traits to establish a division of labor (Amdam et al. 2004, 2006; Page 2013; Page and Amdam 2007). Similarly, queen and larval cues have been modified by natural selection into effective pheromone signals that help coordinate brood care and impact the same fundamental building blocks of reproductive physiology. Queens influence worker behavior via QMP, stimulating retinue behavior (Keeling et al. 2003) and upregulating worker genes tied to nursing (Whitfield et al. 2003). Larvae, similarly dependent on care from the workers, influence worker behavior via brood pheromones, increasing protein foraging required for brood food production (Pankiw et al. 1998; Traynor 2014). Thus, both queen and larval pheromones suppress ovary development and enhance nurse physiology, suggesting that nursing and reproductive physiology are intimately linked as proposed by the reproductive ground plan.

The effects of BEP on honey bee physiology have been well-investigated, but less is known about the priming effects of the volatile young larval pheromone eβ. How eβ interacts with QMP remains unknown. Pheromones are often context specific and may require the natural conditions of the hive to trigger physiological responses; however, studying the effects of pheromones on the physiology of workers in the context of the hive creates unique obstacles due to trophallactic transmission of pheromone signals among nestmates (Korst and Velthuis 1982; Leoncini et al. 2004), the impact of feeding larvae on worker physiology (Amdam et al. 2009), and the impact of the external environment on developmental maturation and resource foraging (Dreller et al. 1999). We thus resolved to study the effects of eβ on the physiology of nurse-aged bees in the laboratory while mimicking the conditions of a natural hive in a controlled cage setting.

In order to test the interactive effects of eβ and QMP on nursing and reproductive physiology of nurse-aged bees (10 days) in a tightly controlled environment, we ran three preliminary experiments to eliminate potential confounding factors of synthetic QMP, diet, and eβ dose on HPG development and ovary activation. We first addressed an earlier controversy (Willis et al. 1990; Winston and Slessor 1998) on the ability of synthetic QMP to significantly suppress ovary activation by comparing the effects of live mated queens, virgin queens, and synthetic QMP on ovary activation (experiment 1). Virgin queens do not emit the full suite of pheromones of a mated queen, lacking emission of eβ and significantly differing in quantities of other pheromone components compared to mated queens (Gilley et al. 2006; Richard et al. 2007). Bees can only activate their HPGs and ovaries with sufficient access to protein-rich food, but an excess of protein increases mortality (Altaye et al. 2010; Pirk et al. 2010). In the hive, newly emerged bees are fed royal jelly by nurses. We hypothesized that royal jelly incorporated into the diet at 10 % could stimulate HPG development in young bees without increasing mortality, substituting for access to nurse bees (experiment 2). Next, we investigated the effects of high versus low eβ dose on HPG development and ovary activation (experiment 3), since brood pheromones have often produced dose-dependent results (Mohammedi et al. 1998; Sagili et al. 2011). Finally, we tested the effects of eβ and QMP in combination on nurse-aged bees, to see if the queen and young larval brood pheromones had interactive effects on HPG development and ovary activation (experiment 4), key components of nursing, and reproductive physiology. Our hypotheses were that (1) QMP would significantly suppress ovary activation compared to controls; (2) 10 % royal jelly would be sufficient to develop HPG without activating ovaries or increasing mortality; (3) the high dose of eβ would significantly stimulate HPG development for nursing and suppress ovary activation; and (4) both eβ and QMP would synergistically suppress ovary activation and enhance HPG development, thus stimulating the development of the nurse bee phenotype primed for caring of her sisters instead of reproduction.

## Materials and methods

### Bees

For each experiment, combs of capped honey bee mature pupae were removed from five wild-type colonies of *A. mellifera ligustica* headed by commercial queens purchased from northern California and placed in an incubator at 34 °C in cages. The following morning, newly emerged bees less than 18 h old were collected, and six replicates were established. Within a single replicate, bees were randomly selected from only two of the five colonies, caged, and the cage was randomly assigned to a treatment group, so that each replicate was composed of two randomly selected genetic families (genotypes). Thus, replicate encompasses genetic variance between and among colonies. One hundred newly emerged bees were paint marked on the thorax according to treatment and placed in an acrylic cage similar in design to the pain cage (Pain 1966) with the addition of a divider that split the cage in half. This divider was either made of single mesh to provide access to nurse bees or was solid. The cages ensured that the pheromones and diet were distributed among all members via trophallaxis and removed additional pheromone exposure from other colony sources. The cages were maintained at 30 ± 3 °C and 35 ± 4 % humidity in individual, disposable incubators assembled from wax-coated cardboard. Cages for each treatment group were kept together in a vented fume hood with a radiant heat source. The bees were fed *ad libitum* with water, queen or royal jelly candy, and pollen paste, replaced every 1–2 days as necessary. Queen candy was made from 80 % powdered sugar and 20 % honey produced by our apiaries in Arizona. Royal jelly candy was made from 10 % royal jelly, 10 % honey, and 80 % powdered sugar on a *w*/*w* basis. Pollen paste was made from frozen pollen pellets (Crockett Honey, Tempe, AZ) ground and mixed with distilled water until it had the consistency of dough.

### Data collection

Bee mortality was recorded daily. After 10 days, the cages of bees were frozen, and for each cage, six to ten bees were randomly selected, dissected, and evaluated for HPG development, total number of ovarioles comprising each ovary and ovary activation. Ovarioles were counted because ovariole number is positively correlated with behavior and ovary activation (Amdam et al. 2004, 2006).

### Dissections

Bees typically transition out of the brood nest and into other in-hive tasks at 10–12 days of age (Rösch 1930; Seeley 1982, 1995; Seeley and Kolmes 1991). Worker HPG reach peak development at 6 days, then typically diminish in size by 15 days of age and atrophy as bees transition to foraging (Deseyn and Billen 2005). As we were interested in the impacts of eβ on nurse bee physiology, dissections were conducted on bees at 10 days of age.

Both HPGs were dissected from the head capsule and placed into a drop of saline (0.25 mol/l NaCl) on a microscope slide. A representative section was examined at 100×. The activity of HPGs is positively correlated with size (Knecht and Kaatz 1990). Numerous globular acini attach to the long, slender main channel of the HPG, and these acini increase in diameter until 6 days of age, when they begin to shrink (Deseyn and Billen 2005). The gland continues to diminish, so that by 15 days of age, when bees typically transition to foraging, their size corresponds to the still undeveloped gland of newly emerged bees (Deseyn and Billen 2005). HPG development was thus rated using an established scale (Hess 1942), which uses the shape and density of the acini as the main criterion for classification and ranks them from by stage of development: (1) atrophied; (2) slightly swollen with noticeable spacing between acini; (3) swollen with small spacing between acini, capable of producing brood food; and (4) fully developed and tightly clustered, channel obscured by acini. Glands were additionally assigned to one of three classes according to lobe morphology (Wegener et al. 2009) as models predict that eβ can accelerate behavioral maturation (Maisonnasse et al. 2010) and we wanted to determine if forager HPG morphology was present in our nurse-aged bees. Class 1, typical of young broodless workers, consists of glands with small acini showing an uneven surface. Class 2, representative of active nurse bees, is composed of medium-sized to large acini with a smooth surface and numerous secretory vesicles, giving them a yellowish color. Class 3 glands, representative of older foragers, consist of large, but slightly pale and translucent lobes. Class 3 was not found among our samples.

Both ovaries were removed from the bees and placed in a drop of saline. The number of ovary filaments (ovarioles) was counted using a 100× dissecting microscope (Zeiss, Jena, Germany). The stage of ovary activation was classified using an established scale (Pernal and Currie 2000); similar to the 4-point scale of Hess (1942) except absence of activation is scored as a 0 instead of 1: 0, no follicle development; 1, slight enlargement; 2, presence of distinct cells leading to swellings and constrictions; 3, egg volume exceeding that of the nutritive follicle; 4, presence of fully formed eggs. For both HPG development and ovary activation, the most developed score of the pair of organs was used for statistical analyses, as occasionally, there were disparities within a bee.

### Treatments

#### Experiment 1: queen comparison

To determine if synthetic QMP was as effective as a live queen in suppressing ovary activation, we compared cages subjected to one of five treatments: (1) mated queen; (2) virgin queen; (3) virgin queen subjected to two successive CO_2_ treatments, which results in oviposition within a few days despite the lack of mating flight (Mackensen 1947); (4) one slow release strip of synthetic QMP (PseudoQueen, formerly known as BeeBoost, Contech Industries, Victoria, British Columbia) attached near the top of the cage using a plastic zip tie to simulate a queen; or (5) control which received no queen or synthetic QMP. The live queens in the first three treatment groups were unconfined and free to interact with the workers as in a natural colony. No comb was included in the cages to prevent egg laying and rearing of larvae.

#### Experiment 2: royal jelly compared to nurse bee environment

To determine if direct access to royal jelly was sufficient to activate HPG development or if newly emerged bees required contact with nurse bees, cages either received 10 % royal jelly (RJ) candy incorporated into queen candy or had access to 100 nurse bees (N) through a single mesh screen. Nurse bees were collected from a comb of open larvae in wild-type colonies, where they were actively engaged in nursing behavior. Each cage also received a synthetic QMP strip as in experiment 1 to mimic in-hive conditions and replicate conditions of future experiments. Since QMP suppresses ovary activation and RJ incorporated into the diet has previously been linked with ovary activation (Altaye et al. 2010; Lin and Winston 1998; Pirk et al. 2010), we included an additional treatment without QMP as a baseline comparison for ovary activation (OA).

#### Experiment 3: high versus low e-beta ocimene dose

Live larvae suppress OA in attending worker bees via larval pheromones, though the effectiveness of pheromones is often dose dependent (Maisonnasse et al. 2009; Mohammedi et al. 1998). To confirm that eβ can suppress OA, we subjected each cage to one of three treatments: (1) low eβ dose of one larval equivalent (Leq)/bee; (2) high eβ dose of 10 Leq/bee; (3) carrier control. Due to the high volatility of eβ and in order to avoid pheromone saturation in the cages, the molecule was mixed with 1-ml paraffin oil, and a similar droplet was used as the control (Maisonnasse et al. 2009). Treatments were supplied in a mesh screened glass Petri dish below the screened floor of the cage; so, bees could not contact the chemicals directly (Maisonnasse et al. 2010). Treatments were replaced daily. Each cage received RJ candy as their carbohydrate source.

#### Experiment 4: eβ and QMP synergy

Pheromones are often context specific and interact with other pheromone components. To determine if eβ and QMP have interactive effects, each cage was subjected to one of four treatments: (1) eβ−/QMP−, (2) eβ−/QMP+, (3) eβ+/QMP−; and (4) eβ+/QMP+. The eβ was supplied at 10 Leq/bee in 1-ml paraffin oil as in experiment 3. The QMP was supplied in a slow release strip of synthetic QMP (PseudoQueen, Contech Industries), as in experiments 1 and 2. Each cage received RJ candy as their carbohydrate source.

### Statistics

Daily mortality was compared using repeated measures MANOVA with replicate and treatment as factors. Total ovarioles, OA, and HPG development were compared using two-way ANOVA with replicate and treatment as factors. Bivariate correlations for total ovarioles, OA, and HPG development were calculated using nonparametric Spearman’s rank correlations. All calculations were performed using JMP Pro 10.0.0 (SAS, Cary, NC).

## Results

### Experiment 1: queen comparison

**Fig. 1:**
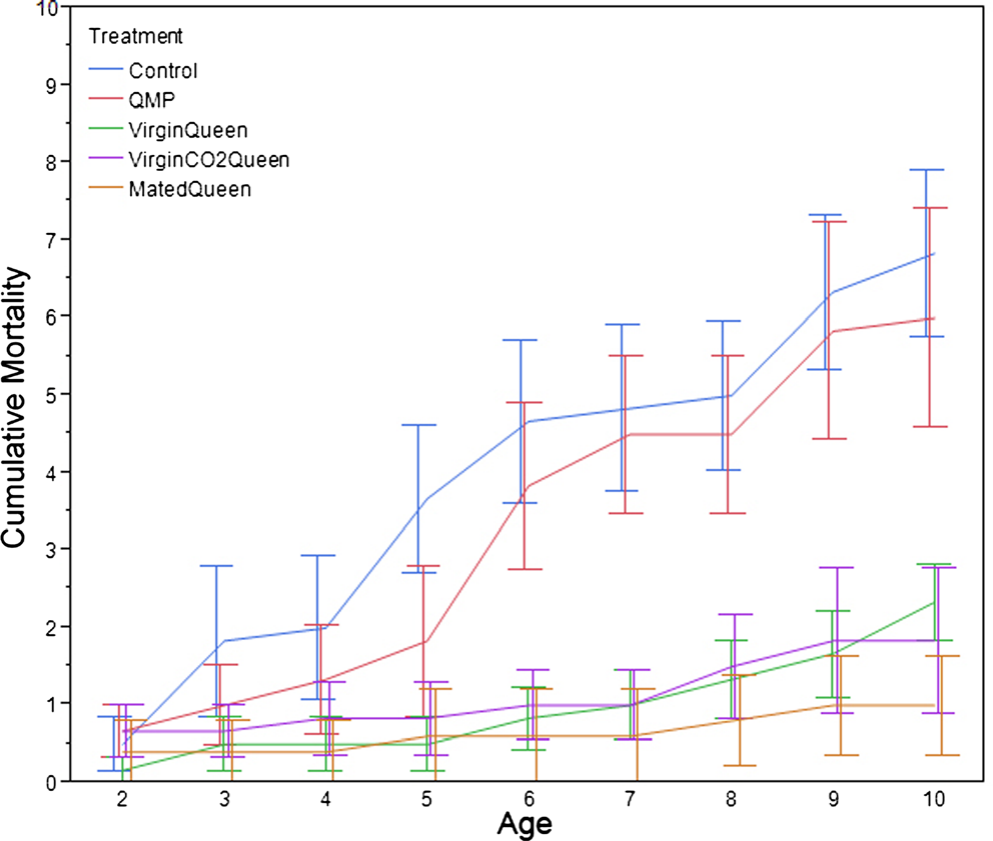
*Experiment 1* queen comparison cumulative mortality (+S.E.) per cage over 10 days. Control (*blue*), QMP (*red*) = synthetic strip of queen mandibular pheromone; VirginQueen (*green*) = virgin queen; Virgin CO2Queen (*purple*) = virgin queen exposed to two treatments of CO_2_; MatedQueen (*tan*) = mated queen

We compared the effects of synthetic QMP and live queens on mortality and ovarian status in caged worker bees. The mated queen in replicate 2 died on day 6 of the experiment, and the cage was excluded from analysis. Mortality was significantly affected by treatment (Fig. 1; *F*_4,20_ = 0.854, *p* = 0.012) and age (*F*_8,13_ = 3.616, *p* = 0.003). Control and QMP cages had significantly higher mortality than the treatments that received a live queen (*t* > 2.50, *p* < 0.013), though mean mortality never exceeded 1 bee/day for any of the treatment groups.

**Fig. 2:**
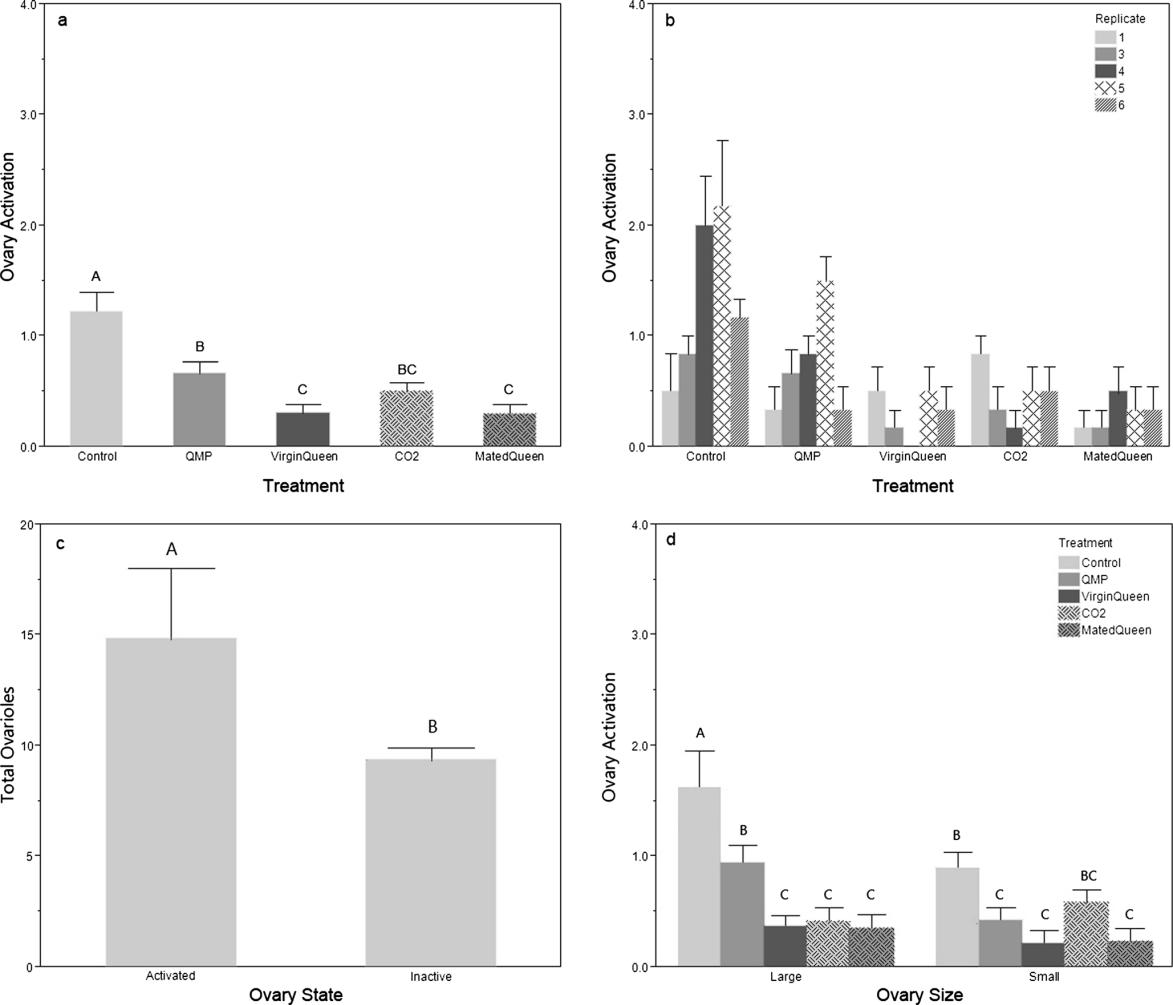
*Experiment 1* queen comparison ovary activation. **a** Mean (+S.E.) ovary activation by treatment; **b** mean (+S.E.) ovary activation by replicate; **c** mean (+S.E.) total ovarioles by ovary state; **d** mean (+S.E.) ovary activation by ovary size and treatment. QMP = synthetic queen mandibular pheromone; CO2 = virgin queen treated 2× with CO_2_; large ovary = (8 or more ovarioles); small ovary (<8 ovarioles). *N* = 180 bees, 36 per treatment, 30 per replicate, 10 bees per cage. *Different letters* indicate significant differences using LSD student *t* tests

OA differed significantly by both treatment (Fig. 2a; *F*_4, 149_ = 15.506, *p* < 0.001) and replicate (Fig. S1; *F*_5,149_ = 4.476, *p* = 0.002), and there was a significant interaction of these two factors (Fig. 2b; replicate 2 excluded; *F*_16,125_ = 2.55, *p* = 0.002). Bees with more ovarioles often activate their ovaries more readily, as seen in this experiment (Fig. 2c; *F*_1,172_ = 5.92, *p* = 0.016). Bees with large ovaries (eight or more ovarioles) had significantly more activated ovaries than bees with small ovaries (*t* = 2.43, *p* = 0.016). Ovary size (large vs small) significantly influenced the effect of treatment on OA (Fig. 2d; *F*_4,164_ = 2.67, *p* = 0.034). Regardless of ovary size, bees in the control group had significantly greater OA compared to the four other queen treatments (*t* > 3.00, *p* < 0.003). In bees with small ovaries, QMP suppressed OA as well as a live queen; however, in bees with large ovaries, QMP was not as effective as a live queen (*t* > 2.40 1.71, *p* < 0.02). Total ovarioles and OA are significantly positively correlated in bees reared in the control (Table 1; *ρ* = 0.430, *n* = 30, *p* = 0.018) environment, but not in any of the treatments with a live queen or with synthetic QMP (Table 1; *ρ* = 0.309, *n* = 30, *p* = 0.097).

**Table 1:** Significant correlations by experiment

Experiment	Treatments	Ovary activation and total ovarioles	Ovary activation and HPG development	Total ovarioles and HPG development
1	C	+	n/a	n/a
	QMP	NS	n/a	n/a
	CO2	NS	n/a	n/a
	VQ	NS	n/a	n/a
	MQ	NS	n/a	n/a
2	RJ	NS	+	NS
	N	+	NS	NS
3	C	NS	NS	NS
	Low eβ	+	NS	NS
	High eβ	+	+	NS
4	eβ−/QMP−	NS	NS	NS
	eβ−/QMP+	+	NS	NS
	eβ+/QMP−	+	+	NS
	eβ+/QMP+	NS	NS	NS

Significant correlations between total ovarioles, ovary activation, and HPG development are given for each of the four experiments that are indicated. Significant correlations are indicated by + or −, depending on relationship. Untested correlations because the HPG were not dissected are indicated by n/a.

### Experiment 2: royal jelly compared to nurse bee environment

In a colony, young bees are fed protein-rich RJ from nurse bees (Crailsheim 1991, 1992), which may impact survivorship and promote development of both the ovaries and HPGs. We investigated the effects of access to nurse bees versus direct access to RJ. Because RJ can stimulate OA and QMP suppresses OA, we included a third treatment group without nurse bees, RJ, or QMP as a baseline comparison for OA. Mortality remained below 1 bee per day, and there was no significant difference in mortality between the RJ and nurse bee (N) treatment groups (*F*_1,5_ = 0.154, *p* = 0.421).

**Fig. 3:**
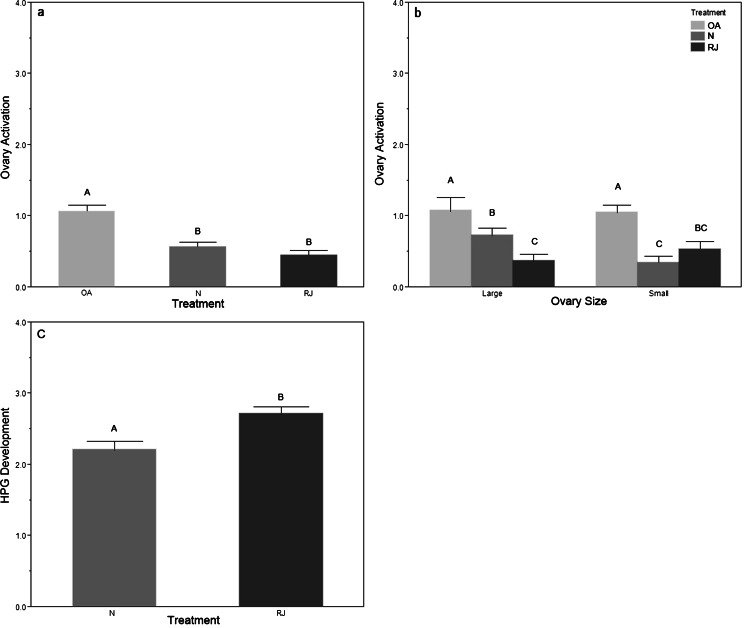
*Experiment 2* access to royal jelly versus nurse bees. **a** Mean (+S.E.) ovary activation by treatment; **b** mean (+S.E.) ovary activation by treatment and ovary size; **c** mean HPG development by treatment. *N* = bees exposed to candy, pollen, QMP, and 100 nurses; RJ = bees exposed to pollen, QMP and 10 % royal jelly incorporated into the queen candy; OA = an ovary activation treatment group without QMP as a baseline comparison. *N* = 180 bees, 60 per treatment, 30 per replicate, 10 bees per cage. *Different letters* indicate significant differences using LSD student *t* tests

OA did not differ between RJ and N treatment groups (*t* = 1.16, *p* = 0.247); there was a significant difference by replicate (*F*_5,114_ = 2.41, *p* = 0.041), but only replicates 1 and 4 were significantly different (*t* = 2.96, *p* = 0.004). The OA treatment group differed significantly from both RJ and N (Fig. 3a; RJ *t* = 5.56, *p* < 0.001; N *t* = 4.51, *p* < 0.001). Ovary size significantly influenced OA in bees exposed to nurses (*t* = 2.80, *p* = 0.007) but had no effect in the two other treatment groups (Fig. 3b).

Nurse-aged bees typically have well-developed HPGs, needed to produce the protein-rich food they feed to larvae. RJ significantly increased HPG development compared to N (Fig. 3c; *t* = 3.69, *p* < 0.001). Replicate had a significant impact on HPG development (*F*_5,108_ = 6.24, *p* < 0.001). HPG development and OA were significantly correlated for bees reared with RJ (*ρ* = 0.259, *n* = 60, *p* = 0.046) but were not significant in bees with access to nurse bees (*ρ* = 0.068, *n* = 60, *p* = 0.604). Total ovarioles and OA were positively correlated in the bees with access to nurse bees (*ρ* = 0.348, *n* = 60, *p* = 0.006).

### Experiment 3: high versus low e-beta ocimene dose

Mortality did not differ significantly by treatment (*F*_2,10_ = 0.725, *p* = 0.066) or age (*F*_8,3_ = 11.078, *p* = 0.134) but varied significantly by replicate (*F*_5,10_ = 1.950, *p* = 0.032).

**Fig. 4:**
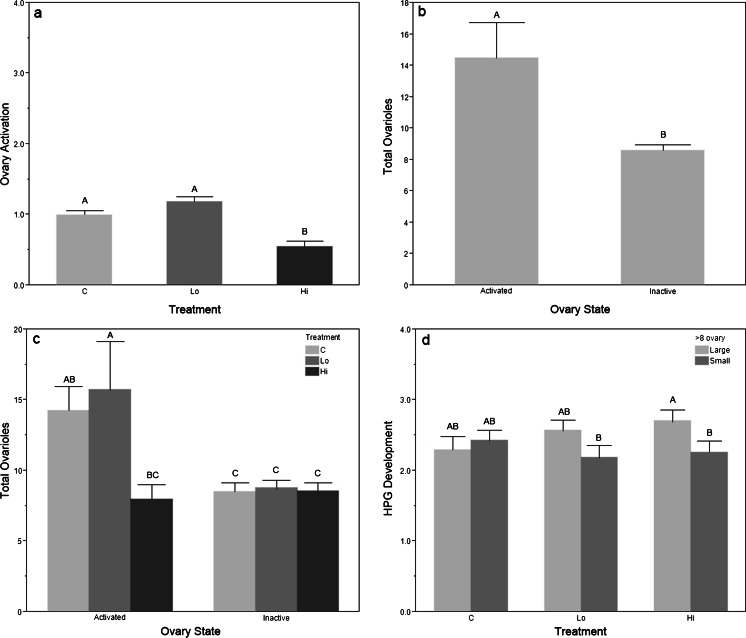
*Experiment 3* eβ dose. **a** Mean (+S.E.) ovary activation by treatment; **b** mean (+S.E.) ovarioles by ovary state; **c** mean (+S.E.) ovarioles by ovary state and treatment; **d** mean (+S.E.) HPG by treatment and ovary size. C = control; Lo = low eβ (1 Leq/bee); Hi = high eβ (10 Leq/bee); activated = one ovary at stage 2 or more; large = 8 or more ovarioles; small = <8 ovarioles; *N* = 180 bees, 60 per treatment, 30 per replicate, 10 bees per cage. *Different letters* indicate significant differences using LSD student *t* tests

Treatment significantly impacted OA (Fig 4a; *F*_2,162_ = 20.73, *p* < 0.001). Bees that received the high eβ dose of 10 Leq/bee had significantly fewer developing oocytes than bees in the control group or receiving the low dose of 1 Leq/bee (*t* > 4.40, *p* < 0.001). As above, bees with more ovarioles had significantly more activated ovaries than bees with fewer ovarioles (Fig. 4b; *t* = 3.62, *p* < 0.001). Bees with activated ovaries had significantly more ovarioles in both the control (Fig. 4c; *t* = 2.38, *p* = 0.035) and low eβ (*t* = 3.59, *p* < 0.001) treatment groups, but not in the high eβ group (*t* = 0.20, *p* = 0.844). In bees exposed to the high dose, 50 % of bees had at least one ovary with OA at or above stage 1 (slight ovariole swelling) and 3 % at or above stage 2 compared to 97 and 18 %, respectively, in the low dose and 92 and 7 % in the control.

**Fig. 5:**
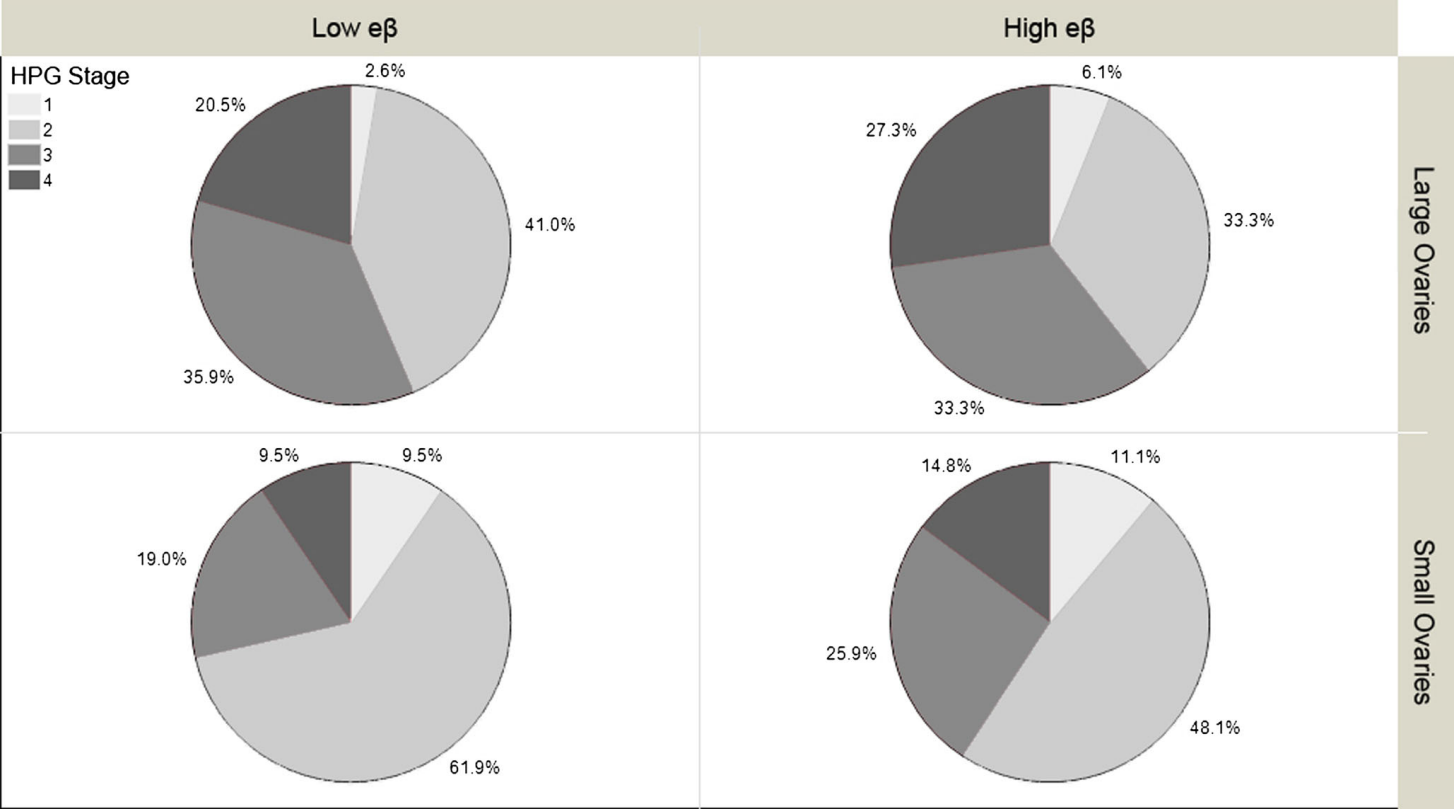
*Experiment 3* eβ dose. HPG development by ovary size for bees treated with low eβ (*left*) and high eβ (*right*). HPG stages 1 and 2 (*light grays*) are incapable of nursing, while stages 3 and 4 (*dark greys*) are capable of nursing. Large (*top row*) = 8 or more ovarioles; small (*bottom row*) = <8 ovarioles

There was no significant effect of treatment on HPG development, indicating that eβ did not increase HPG development compared to controls (*F*_2,162_ = 1.06, *p* = 0.348); HPG development differed across replicates (*F*_5,162_ = 4.34, *p* = 0.001). In the groups treated with eβ, bees with large ovaries had significantly more developed HPG than bees with small ovaries (*t* = 2.59, *p* = 0.01), but there was no difference in the control group (*t* = 0.56, *p* = 0.57), see Fig. 4d. In bees with large ovaries treated with high eβ, 60.6 % had developed HPG glands capable of nursing (stage 3 or 4) compared to 40.7 % of the bees with small ovaries. A similar trend was seen with the low eβ dose, where 56.4 % of bees with large ovaries had well-developed HPG, compared to 28.5 % with small ovaries (see Fig. 5). Thus, total ovarioles were significantly correlated with OA for bees reared in the low eβ (*ρ* = 0.432, *n* = 60, *p* < 0.001) and high eβ environment (*ρ* = 0.341, *n* = 60, *p* = 0.008), but not in the control group (total ovarioles *ρ* = 0.212, *n* = 60, *p* = 0.104). Pairwise correlations show that OA and HPG development are positively correlated in the high eβ environment (*r* = 0.270, *n* = 60, *p* = 0.037), but the correlation is not significant when converted to nonparametric ranks (*ρ* = 0.243, *n* = 60, *p* = 0.062).

### Experiment 4: eβ and QMP synergy

Having established that both QMP and the high dose of eβ significantly suppresses OA compared to controls, we tested the interactive effects of eβ and QMP. Mortality did not differ significantly by treatment (*F*_3,15_ = 0.182, *p* = 0.460) but differed significantly by replicate (Fig. S3; *F*_5,15_ = 1.107, *p* < 0.032) and age (*F*_7,9_ = 3.585, *p* < 0.019).

**Fig. 6:**
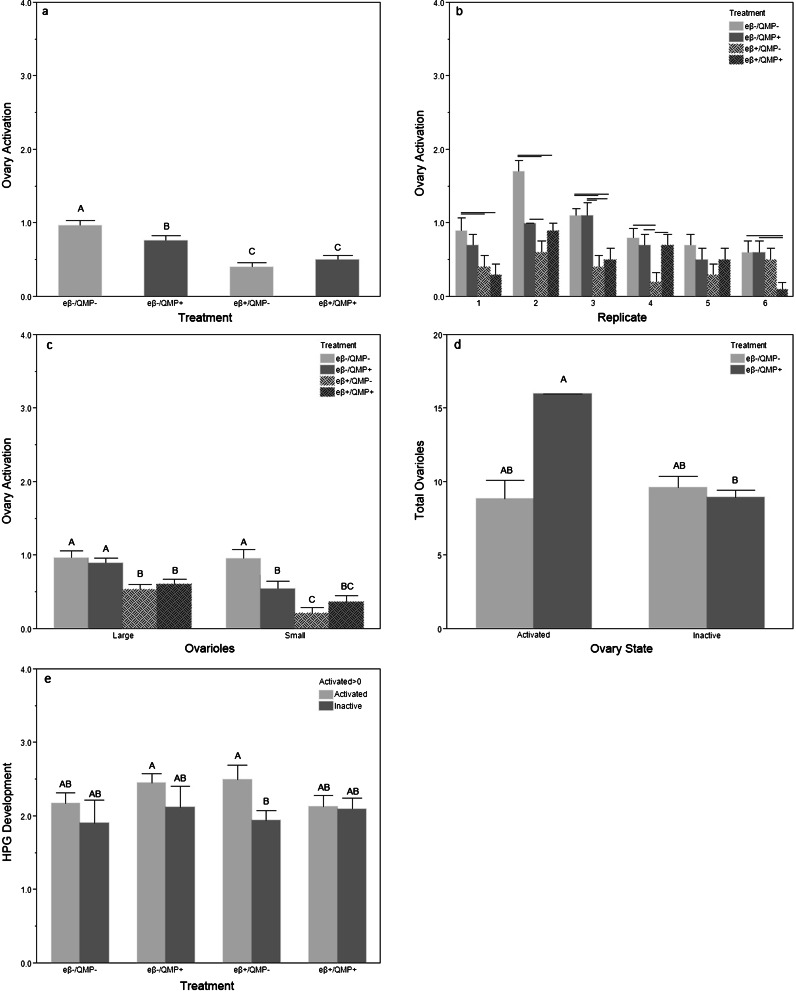
*Experiment 4* eβ and QMP interaction. **a** Mean (+S.E.) ovary activation by treatment; **b** mean (+S.E.) ovary activation by treatment and replicate; **c** mean (+S.E.) ovary activation by ovary size; **d** mean (+S.E.) ovarioles by ovary state and treatment; **e** mean (+S.E.) HPG by treatment and ovary state. eβ = e-beta; QMP = synthetic queen mandibular pheromone, large = 8 or more ovarioles; small = <8 ovarioles; activated = one ovary at stage 2 or more; for 6e) activated >0 = one ovary at stage 1 or more; *N* = 240 bees, 60 per treatment, 30 per replicate, 10 bees per cage. *Different letters or connecting bars* indicate significant differences

Total ovariole number per bee did not differ by treatment (*F*_3,216_ = 0.30, *p* = 0.822) but varied significantly by replicate (*F*_5,216_ = 2.53, *p* = 0.030). Replicate 3 had significantly more ovarioles than replicates 1, 4, 5, and 6. Treatment significantly impacted OA (Fig. 6a; *F*_3,216_ = 17.73, *p* < 0.001). Bees reared with eβ had significantly less-developed ovaries than bees reared without eβ (control *t*_216_ > 5.39, *p* < 0.001; QMP *t*_216_ > 3.08, *p* < 0.003). The bees reared with QMP and no eβ had significantly less-developed ovaries than control bees reared without either pheromone (*t*_216_ = 2.31, *p* = 0.022). However, bees reared with only QMP had significantly more developed ovaries than bees exposed to eβ (eβ alone *t*_216_ = 4.24, *p* < 0.001; eβ and QMP *t*_216_ = 3.08, *p* = 0.002), indicating that eβ is more effective at suppressing OA than QMP. In the control group, 82 % of bees had at least stage 1 OA in one ovary, compared to 73 % of the bees exposed only to QMP, 40 % of the bees exposed to only eβ and 50 % of the bees exposed to both eβ and QMP. OA also differed by replicate (*F*_5,216_ = 17.023, *p* < 0.001), seemingly a consequence of differences in total ovarioles as replicates 2 and 3 had the most total ovarioles combined with the most activated ovaries. There was a significant interaction of treatment and replicate (Fig. 6b; *F*_15,216_ = 1.74, *p* = 0.046). Once again, bees with large ovaries (eight or more ovarioles) had significantly more active ovaries than bees with small ovaries (*t*_228_ = 2.72, *p* = 0.007), and ovary size was a significant factor of OA in bees treated with only one of the two pheromones (Fig. 6c), but not in bees treated with both or in the control group. There were no bees with stage 2 activation in at least one ovary in either eβ group. In the QMP-treated group, only bees with significantly more ovarioles were able to activate their ovaries at stage 2 or above (Fig. 6d; *t*_116_ = 2.13, *p* = 0.035), while ovariole number did not influence OA in the control group (*t*_116_ = 0.46, *p* = 0.649).

HPG stage development did not differ significantly by treatment (*F*_3,216_ = 1.06, *p* = 0.365) or replicate (*F*_5,216_ = 1.41, *p* = 0.223). Bees with OA above stage 1 had significantly more developed HPG than bees with inactive ovaries across all treatments (*F*_1,232_ = 4.279, *p* = 0.024), and this effect was significant within the eβ+/QMP− treatment group (Fig. 6e; *t* = 2.291, *p* = 0.027), where 50 % of bees with activated ovaries had HPG capable of nursing (stage 3 or 4) compared to 19.5 % of bees with inactive ovaries. Thus, HPG development and OA were significantly correlated for bees reared in the eβ+/QMP− (*ρ* = 0.295, *n* = 60, *p* = 0.022), but not in any of the other groups. Total ovarioles and OA also correlated significantly in the eβ+/QMP− (*ρ* = 0.323, *n* = 60, *p* = 0.012) and the eβ−/QMP+ (*ρ* = 0.443, *n* = 60, *p* < 0.001) environments, but not in the other treatment groups.

## Discussion

Our results demonstrate how social insect pheromone communication is defined by complexity, context, and dose (Alaux et al. 2010; Slessor et al. 2005). Throughout our experiments, QMP significantly suppressed OA in worker bees compared to controls (Figs. 2a and 6a), as did the eβ pheromone of young larvae (Figs. 4a and 6a). Our results also show that eβ had significant effects on the reproductive and nursing physiology of worker bees, so that bees with more ovarioles had increased OA (Figs. 4b–c and 6d) and increased HPG development (Figs. 4d, 5, and 6e). This trend of HPG development and OA in bees with more ovarioles started to appear in the low eβ-treated bees (Fig. 4d) and was significant and consistent in bees treated with high eβ (Figs. 4d and 6e). The correlation disappears in the presence of QMP (Fig. 6e and Table 1), suggesting that QMP and eβ interact to suppress OA in bees with more ovarioles.

### Replicate effects

Within a single replicate, bees were randomly selected from two of the five donor colonies to minimize within cage variance. We did not prescreen colonies for ovariole number or colony wide OA, both of which vary genetically and influence behavior (reviewed in Page 2013). Replicate thus encompasses both individual cage and genetic differences. Replicate frequently proved a significant factor in the experiments, suggesting that genotype may influence individual response thresholds to pheromones, as has been demonstrated in other experiments (Amdam et al. 2009; Pankiw and Page 1999, 2001). While there were frequently significant differences between replicates, the replicates typically followed the same trend and only interacted with the treatment group when indicated (Figs. 2b and 5b).

### Mortality

Although daily mortality remained low (<1 bee/day across all experiments), the presence of a live queen significantly reduced mortality compared to synthetic QMP or control groups (Fig. 1). This suggests that live queens enhance survival compared to synthetic QMP, perhaps by reducing overall stress, reducing reproductive competition among workers, and adding to group cohesion by their presence.

### Ovary activation

Egg laying in insects involves two distinct processes, the production of the egg yolk proteins from the egg yolk precursor vitellogenin (Vg) and the incorporation of these proteins into eggs, followed by the physical oviposition of developed eggs. QMP and eβ appear to act on different components of the reproductive physiology in honey bee workers, with the former suppressing OA in bees with fewer ovarioles (Fig. 1d), while the latter suppresses OA across all bees at the higher dose of ten larval equivalents per bees (Figs. 4c and 6c).

When queens are present in a colony, there are very low incidences of worker egg laying, though some level of OA is always present (Page and Erickson 1988). In queenless colonies, some workers become the dominant egg layers and act as false queens (Sakagami 1958) that attract a queen retinue and suppress physical egg laying in other workers by emitting a queen-like mandibular pheromone (Crewe and Velthuis 1980). When these false queens are removed, the other workers immediately begin laying eggs (Page and Robinson 1994; Robinson et al. 1990), illustrating that queen pheromones suppress egg laying but do not suppress OA (Jay and Nelson 1973) as well as larval pheromones (this experiment). Workers with activated ovaries are often found in queenright colonies that lack brood (Jay 1972) or when the brood nest is diminished just prior to swarming (Kropacova and Haslbachova 1970).

Our queen comparison experiment showed that synthetic QMP significantly suppresses OA compared to controls, though live queens are more effective than QMP in suppressing OA in bees with more ovarioles (Fig. 2d). Bees had continual access to QMP, frequently clustering over the synthetic strip. Throughout our experiments, bees with more ovarioles were most likely to activate their ovaries (Figs. 2c, 3b, 4b, c, and 6c, d), as has been shown previously (Amdam et al. 2006; Graham et al. 2011; Linksvayer et al. 2009; Page and Amdam 2007; Page et al. 2006, 2012; Tsuruda et al. 2008; Wang et al. 2010). Ovariole number is a recognized marker of reproductive potential in honey bees (Makert et al. 2006; Tanaka and Hartfelder 2004) demonstrating that workers with the most ovarioles and thus greatest reproductive potential are most likely to escape ovary suppression.

The inability of QMP to suppress OA as strongly as a live queen suggests that more factors are involved in reproductive suppression. Only live queens, who emit multiple pheromones (QMP, Dufour’s gland, and tergal pheromones), can fully suppress OA in workers, though both live queens and QMP disassociated total ovarioles from OA (Table 1) (Hoover et al. 2003; Katzav-Gozansky 2006; Slessor et al. 2005; Willis et al. 1990). This difference between QMP and live queens has been postulated to be a sign of a queen “control” and a continuing evolutionary arms race over male reproduction (Katzav-Gozansky 2006). Alternatively, the multicomponent pheromone could represent an honest signal of queen fecundity linked to reproductive state that encourages worker “cooperation” and informs the colony when the queen starts to fail (Kocher and Grozinger 2011).

QMP suppresses juvenile hormone (JH) biosynthesis (Robinson et al. 1992). In honey bees, JH and Vg are normally coregulated in a double-repressor relationship (Amdam and Omholt 2003; Ihle et al. 2010); high circulating titers of JH suppress production of Vg and conversely high titers of Vg suppress JH. Since QMP suppresses JH production, these low JH titers in turn augment Vg titers, stimulating production of the egg yolk precursor required for OA.

In the absence of QMP, the eβ high dose of 10 Leq/bee significantly suppressed OA (Fig. 4a) as seen in previous experiments (Maisonnasse et al. 2009), paralleling the effects of live larvae, which inhibit worker OA (Jay 1972; Jay and Jay 1976). A queenless hive can survive by rearing a replacement queen from larvae present in the colony (Hatch et al. 1999). However, workers made queenless refrain from rearing an emergency queen for 24 h in the presence of eggs and young larvae but start rearing queens immediately when only older larvae (3rd–5th larval instar) are available (Pettis et al. 1997), indicating that the eggs and/or young larvae provide a fecundity signal that fades after 24 h in the absence of a queen. The low dose of 1 Leq/bee of eβ had no effect on ovary suppression.

Our eβ and QMP synergy experiment (experiment 4) demonstrates that eβ is more effective than synthetic QMP at suppressing OA, and there is no apparent interactive effect on OA between the two pheromones (Fig. 6a), at least not at 10 days of age. Our results confirm that both young (current results) and old larval brood pheromones are very effective in suppressing OA and worker reproduction (Arnold et al. 1994; Maisonnasse et al. 2009; Mohammedi et al. 1998). Just as live queens and QMP resulted in a disassociation between total ovarioles and OA (Table 1, experiment 1), suggesting suppression of OA regardless of the underlying reproductive physiology, a similar disassociation occurred in our eβ and QMP synergy experiment in bees exposed to both brood and queen pheromones (Table 1, experiment 4).

Throughout all of our experiments, we saw low levels of OA at 10 days of age, with mean OA never exceeding stage 1, classified as slight swelling at the top of the ovariole. Control bees consistently had 80 % or more bees with stage 1 OA and 8–15 % of bees with vitellogenic ovaries. Bees typically transition out of the brood nest and into other in-hive tasks at 10–12 days of age (Rösch 1930; Seeley 1982, 1995; Seeley and Kolmes 1991). As we were interested in the impacts of eβ on nurse bee physiology, we limited the duration of our cage trials to 10 days. Thus, the possibility remains that synergy between QMP and eβ on suppression of worker reproduction could occur in more prolonged experiments, with eβ suppressing OA and QMP stopping egg laying, although no significant differences or trends were evident between eβ+/QMP− and eβ+/QMP+ at 10 days.

### HPG development

Incorporating RJ into the diet at 10 % was more effective than access to nurse bees in stimulating HPG development, resulting in almost twice as many bees with well-developed HPGs, classified as stage 3 or 4. Adequate nutrition is essential for both HPG development and OA (Haydak 1970; Hoover et al. 2006; Hrassnigg and Crailsheim 1998).

Bees that experienced the high eβ environment developed their HPG significantly more when they had large ovaries compared to small ovaries (Fig. 4d). This suggests that worker bees may be more strongly influenced to activate their HPG for larval feeding, if they are predisposed to nursing by possessing more ovariole filaments. Additionally, they may be more prone to activate their ovaries if they have no larvae to receive the brood food, thus repurposing the Vg from their HPG (Amdam and Omholt 2003; Seehuus et al. 2007) into their ovaries to produce eggs. Early OA in bees with more ovarioles is correlated with higher titers of Vg that subsequently drop. It is hypothesized that ovariole number and the dynamics of Vg expression influence the onset of foraging and foraging behavior (Ihle et al. 2010; Nelson et al. 2007; Page 2013) in *A. mellifera*, except for subspecies *Apis mellifera capensis* (Roth et al. 2014), where bees with more ovarioles do not forage precociously. However, “the reproductive control system in *A. m. capensis* is unique when compared with other honeybee subspecies,” (Zheng et al. 2010) and thus should not be used to dismiss the coupling of reproductive and nursing physiology in all other *A. mellifera*. In our experiments, eβ appears to have greater effects on bees with more ovarioles, priming them for both larval care and protein-rich pollen foraging, behavior that supports the nutritional development of the young larvae emitting the pheromone.

## Conclusion

Our current results reinforce the reproductive ground plan hypothesis that postulates that ancestral reproductive physiology was coopted and used to regulate foraging behavior (Amdam et al. 2004, 2006; Page and Amdam 2007; Page et al. 2006). Early nutritional differences in larval development lead to variation in worker ovariole number (Leimar et al. 2012; Wang et al. 2014) and thus may contribute to differential response thresholds to eβ priming. In field trials, eβ both releases and primes bees toward pollen collection (Traynor 2014), a pollen-foraging bias predicted by the reproductive ground plan hypothesis (Page 2013; Page and Amdam 2007; Page et al. 2006). Our results thus suggest that eβ impacts worker physiology tied to maternal traits differentially in predisposed bees that possess more ovariole filaments at both life stages of worker development: During early adult life, eβ improves nursing physiology by stimulating HPG development. After the transition to foraging, eβ biases bees toward pollen collection to provide protein for the developing brood nest.

Young adult bees actively tending the brood nest typically have the most developed HPG in a colony. The queen spends the majority of her time in the brood nest laying eggs in the vicinity of these nurse bees; thus, the nurse bees have the greatest opportunity for interaction with the queen. When the queen is absent, QMP is not present, and when her reproductive potential starts to fail, there is a reduction of brood and thus a diminishing eβ signal. At this point, the nurse bees may detect the changes and reroute Vg from their HPG to their own ovaries for activation and an opportunity for reproduction (Bier 1954, 1958), as seen throughout our experiments in the control bees raised without eβ or QMP.

Our experimental results illustrate that pheromones in social insects provide complex signals that must be interpreted in context-dependent circumstances and are strongly impacted by individual worker physiology. Honey bee chemical communication has dynamic properties and functions as a property of a complex system (Pankiw 2004). QMP and eβ play important roles in honey bee society as both primer and releaser pheromones that change putative response thresholds to different stimuli by altering reproductive physiology and interacting with innate response thresholds of different genotypes. The young larval pheromone eβ suppresses OA across all bees and activates HPG predominantly in bees that have become tuned to nursing because of their heightened number of ovarioles. Larval eβ primes these more responsive workers to enhance larval provisioning by increasing HPG development to produce more brood food and by activating their ovaries, tuning those workers to bias later foraging toward pollen collection. Additional field trials that examine the role of eβ on honey bee physiology in the context of the hive are needed to complement our current results, as well as experiments that probe the interactions between young and old larval pheromones in concert with QMP.

## Electronic supplementary material

Below is the link to the electronic supplementary material.

Supplemental Fig. S1 Experiment 2: access to royal jelly vs. nurse bee mean (+S.E.) ovary activation by replicated. N = 180 bees, 60 per treatment, 30 per replicate, 10 bees per cage. Different letters indicate significant differences using LSD student t tests. (JPEG 60 kb)

Supplemental Fig. S2 Experiment 3: eβ dose cumulative mortality (+S.E.) per cage over 10 days. N = 180 bees, 60 per treatment, 30 per replicate, 10 bees per cage. (JPEG 136 kb)

Supplemental Fig. S3 Experiment 4: eβ & QMP cumulative mortality (+S.E.) per cage over 10 days. N = 240 bees, 60 per treatment, 30 per replicate, 10 bees per cage. (JPEG 137 kb)

## References

[CR1] Alaux C, Maisonnasse A, Le Conte Y (2010). Pheromones in a superorganism: from gene to social regulation. Vitam Horm.

[CR2] Altaye SZ, Pirk CWW, Crewe RM, Nicolson SW (2010). Convergence of carbohydrate-biased intake targets in caged worker honeybees fed different protein sources. J Exp Biol.

[CR3] Amdam GV, Omholt SW (2003). The hive bee to forager transition in honeybee colonies: the double repressor hypothesis. J Theor Biol.

[CR4] Amdam GV, Norberg K, Fondrk MK, Page RE (2004). Reproductive ground plan may mediate colony-level selection effects on individual foraging behavior in honey bees. Proc Natl Acad Sci U S A.

[CR5] Amdam GV, Csondes A, Fondrk MK, Page RE (2006). Complex social behaviour derived from maternal reproductive traits. Nature.

[CR6] Amdam GV, Rueppell O, Fondrk MK, Page RE, Nelson CM (2009). The nurse’s load: early-life exposure to brood-rearing affects behavior and lifespan in honey bees (*Apis mellifera*). Exp Gerontol.

[CR7] Arnold G, Le Conte Y, Trouiller J, Hervert H, Chappe B, Masson C (1994). Inhibition of worker honeybee ovaries development by a mixture of fatty acid esters from larvae. C R Acad Sci.

[CR8] Bier K (1954) Über den Einfluss der Königin auf die arbeiterinnen Fertilität im Ameisenstaat. Insectes Soc 1:7–19

[CR9] Bier K (1958). Die regulation der sexualitaet in den insektenstaaten. Ergebnisse der Biologie.

[CR10] Crailsheim K (1991). Interadult feeding of jelly in honeybee (*Apis mellifera* L.) colonies. J Comp Physiol B.

[CR11] Crailsheim K (1992). The flow of jelly within a honeybee colony. J Comp Physiol B.

[CR12] Crewe RM, Velthuis HHW (1980). False queens: a consequence of mandibular gland signals in worker honeybees. Naturwissenschaften.

[CR13] De Groot A, Voogd S (1954). On the ovary development in queenless worker bees (*Apis mellifica* L.). Experientia.

[CR14] Deseyn J, Billen J (2005). Age-dependent morphology and ultrastructure of the hypopharyngeal gland of *Apis mellifera* workers (Hymenoptera, Apidae). Apidologie.

[CR15] Dreller C, Page RE, Fondrk MK (1999). Regulation of pollen foraging in honeybee colonies: effects of young brood, stored pollen, and empty space. Behav Ecol Sociobiol.

[CR16] Free JB (1987). Pheromones of social bees.

[CR17] Gilley DC, Degrandi-Hoffman G, Hooper JE (2006). Volatile compounds emitted by live European honey bee (*Apis mellifera* L.) queens. J Insect Physiol.

[CR18] Graham AM, Munday MD, Kaftanoglu O, Page RE, Amdam GV, Rueppell O (2011). Support for the reproductive ground plan hypothesis of social evolution and major QTL for ovary traits of Africanized worker honey bees (*Apis mellifera* L.). BMC Evol Biol.

[CR19] Hatch S, Tarpy D, Fletcher D (1999). Worker regulation of emergency queen rearing in honey bee colonies and the resultant variation in queen quality. Insectes Soc.

[CR20] Haydak MH (1970). Honey bee nutrition. Annu Rev Entomol.

[CR21] Hess G (1942). Über den einfluss des weisellosigkeit und des fruchtbarkeitsvitamins E auf die ovarien der bienenarbeiterin. Beih Schweiz Bienenzeitung.

[CR22] Hoover SE, Keeling CI, Winston ML, Slessor KN (2003). The effect of queen pheromones on worker honey bee ovary development. Naturwissenschaften.

[CR23] Hoover SE, Higo HA, Winston ML (2006). Worker honey bee ovary development: seasonal variation and the influence of larval and adult nutrition. J Comp Physiol B.

[CR24] Hrassnigg N, Crailsheim K (1998). Adaptation of hypopharyngeal gland development to the brood status of honeybee (*Apis mellifera* L.) colonies. J Insect Physiol.

[CR25] Huang Z, Otis GW (1989). Factors determining hypopharyngeal gland activity of worker honey bees (*Apis mellifera* L.). Insectes Soc.

[CR26] Huang Z, Otis G, Teal PEA (1989). Nature of brood signal activating the protein synthesis of hypopharyngeal gland in honey bees, *Apis mellifera* (Apidae:Hymenoptera). Apidologie.

[CR27] Ihle KE, Page RE, Frederick K, Fondrk MK, Amdam GV (2010). Genotype effect on regulation of behaviour by vitellogenin supports reproductive origin of honeybee foraging bias. Anim Behav.

[CR28] Jay S (1970). The effect of various combinations of immature queen and worker bees on the ovary development of worker honeybees in colonies with and without queens. Can J Zool.

[CR29] Jay S (1972). Ovary development of worker honeybees when separated from worker brood by various methods. Can J Zool.

[CR30] Jay S, Jay DH (1976). The effect of various types of brood comb on the ovary development of worker honeybees. Can J Zool.

[CR31] Jay S, Nelson E (1973). The effects of laying worker honeybees (*Apis mellifera* L.) and their brood on the ovary development of other worker honeybees. Can J Zool.

[CR32] Kaatz HH, Hildebrandt H, Engels W (1992) Primer effect of queen pheromone on juvenile hormone biosynthesis in adult worker honey bees. J Comp Physiol B 162:588–592

[CR33] Katzav-Gozansky T (2006) The evolution of honeybee multiple queen pheromones-a consequence of a queen-worker arms race. Braz J Morphol Sci 23:287–294

[CR34] Keeling CI, Slessor KN, Higo HA, Winston ML (2003). New components of the honey bee (*Apis mellifera* L.) queen retinue pheromone. Proc Natl Acad Sci U S A.

[CR35] Knecht D, Kaatz HH (1990). Patterns of larval food production by hypopharyngeal glands in adult worker honey bees. Apidologie.

[CR36] Kocher S, Grozinger C (2011). Cooperation, conflict, and the evolution of queen pheromones. J Chem Ecol.

[CR37] Korst P, Velthuis H (1982). The nature of trophallaxis in honeybees. Insectes Soc.

[CR38] Kropacova S, Haslbachova H (1970). The development of ovaries in worker honeybees in queenright colonies examined before and after swarming. J Apic Res.

[CR39] Le Conte Y, Hefetz A (2008). Primer pheromones in social hymenoptera. Annu Rev Entomol.

[CR40] Le Conte Y, Sreng L, Trouiller J (1994). The recognition of larvae by worker honeybees. Naturwissenschaften.

[CR41] Le Conte Y, Sreng L, Poitout SH (1995). Brood pheromone can modulate the feeding behavior of *Apis mellifera* workers. J Econ Entomol.

[CR42] Le Conte Y, Mohammedi A, Robinson GE (2001). Primer effects of a brood pheromone on honeybee behavioural development. Proc Biol Sci.

[CR43] Leimar O, Hartfelder K, Laubichler MD, Page RE (2012). Development and evolution of caste dimorphism in honeybees-a modeling approach. Ecol Evol.

[CR44] Leoncini I (2004). Regulation of behavioral maturation by a primer pheromone produced by adult worker honey bees. Proc Natl Acad Sci U S A.

[CR45] Lin H, Winston ML (1998). The role of nutrition and temperature in the ovarian development of the worker honey bee (*Apis mellifera*). Can Entomol.

[CR46] Linksvayer TA, Rueppell O, Siegel A, Kaftanoglu O, Page RE, Amdam GV (2009). The genetic basis of transgressive ovary size in honeybee workers. Genetics.

[CR47] Linksvayer TA, Kaftanoglu O, Akyol E, Blatch S, Amdam GV, Page RE (2011). Larval and nurse worker control of developmental plasticity and the evolution of honey bee queen-worker dimorphism. J Evol Biol.

[CR48] Mackensen O (1947). Effect of carbon dioxide on initial oviposition of artificially inseminated and virgin queen bees. J Econ Entomol.

[CR49] Maisonnasse A (2009). A scientific note on E-β-ocimene, a new volatile primer pheromone that inhibits worker ovary development in honey bees. Apidologie.

[CR50] Maisonnasse A, Lenoir JC, Beslay D, Crauser D, Le Conte Y (2010). E-beta-ocimene, a volatile brood pheromone involved in social regulation in the honey bee colony (*Apis mellifera*). PLoS One.

[CR51] Makert G, Paxton R, Hartfelder K (2006). Ovariole number—a predictor of differential reproductive success among worker subfamilies in queenless honeybee (*Apis mellifera* L.) colonies. Behav Ecol Sociobiol.

[CR52] Mohammedi A, Crauser D, Paris A, Le Conte Y (1996). Effect of a brood pheromone on honeybee hypopharyngeal glands. C R Acad Sci III.

[CR53] Mohammedi A, Paris A, Crauser D, Le Conte Y (1998). Effect of aliphatic esters on ovary development of queenless bees (*Apis mellifera L*.). Naturwissenschaften.

[CR54] Nelson CM, Ihle KE, Fondrk MK, Page RE, Amdam GV (2007). The gene vitellogenin has multiple coordinating effects on social organization. PLoS Biol.

[CR55] Page RE (2013). The spirit of the hive: the mechanisms of social evolution.

[CR56] Page RE, Amdam GV (2007). The making of a social insect: developmental architectures of social design. Bioessays.

[CR57] Page RE, Erickson EH (1988). Reproduction by worker honey bees (*Apis mellifera* L.). Behav Ecol Sociobiol.

[CR58] Page RE, Robinson G (1994). Reproductive competition in queenless honey bee colonies (*Apis mellifera* L.). Behav Ecol Sociobiol.

[CR59] Page RE, Scheiner R, Erber J, Amdam GV (2006). 8. The development and evolution of division of labor and foraging specialization in a social insect (*Apis mellifera* L.). Curr Top Dev Biol.

[CR60] Page RE, Rueppell O, Amdam GV (2012). Genetics of reproduction and regulation of honeybee (*Apis mellifera* L.) social behavior. Annu Rev Genet.

[CR61] Pain J (1966). Nouveau modèle de cagettes expérimentales pour le mainten d’abeilled en captivité. Ann Abeille.

[CR62] Pankiw T (2004). Cued in: honey bee pheromones as information flow and collective decision-making. Apidologie.

[CR63] Pankiw T, Page RE (1999). The effect of genotype, age, sex, and caste on response thresholds to sucrose and foraging behavior of honey bees (*Apis mellifera* L.). J Comp Physiol A.

[CR64] Pankiw T, Page RE (2001). Genotype and colony environment affect honeybee (*Apis mellifera* L.) development and foraging behavior. Behav Ecol Sociobiol.

[CR65] Pankiw T, Page RE, Fondrk MK (1998). Brood pheromone stimulates pollen foraging in honey bees (*Apis mellifera*). Behav Ecol Sociobiol.

[CR66] Pernal S, Currie R (2000). Pollen quality of fresh and 1-year-old single pollen diets for worker honey bees (*Apis mellifera* L.). Apidologie.

[CR67] Peters L, Zhu-Salzman K, Pankiw T (2010). Effect of primer pheromones and pollen diet on the food producing glands of worker honey bees (*Apis mellifera* L.). J Insect Physiol.

[CR68] Pettis JS, Higo HA, Pankiw T, Winston ML (1997). Queen rearing suppression in the honey bee—evidence for a fecundity signal. Insectes Soc.

[CR69] Pickett JA, Williams I, Martin AP, Smith MC (1980). Nasonov pheromone of the honey bee, *Apis mellifera* L. J Chem Ecol.

[CR70] Pirk CWW, Boodhoo C, Human H, Nicolson S (2010). The importance of protein type and protein to carbohydrate ratio for survival and ovarian activation of caged honeybees (*Apis mellifera scutellata*). Apidologie.

[CR71] Richard F, Tarpy DR, Grozinger CM (2007). Effects of insemination quantity on honey bee queen physiology. PLoS One.

[CR72] Robinson G, Page RE, Fondrk MK (1990). Intracolonial behavioral variation in worker oviposition, oophagy, and larval care in queenless honey bee colonies. Behav Ecol Sociobiol.

[CR73] Robinson GE, Strambi C, Strambi A, Huang Z (1992). Reproduction in worker honey bees is associated with low juvenile hormone titers and rates of biosynthesis. Gen Comp Endocrinol.

[CR74] Rösch GA (1930) Untersuchungen über die Arbeitsteilung im Bienenstaat. 2. Teil: Die Tätigkeiten der Arbeitsbienen unter experimentell veränderten Bedingungen. Springer-Verlag, Berlin doi:10.1007/978-3-662-41225-1

[CR75] Roth KM, Beekman M, Allsopp MH, Goudie F, Wossler TC, Oldroyd BP (2014). Cheating workers with large activated ovaries avoid risky foraging. Behav Ecol.

[CR76] Sagili RR, Pankiw T (2009). Effects of brood pheromone modulated brood rearing behaviors on honey bee (*Apis mellifera* L.) colony growth. J Insect Behav.

[CR77] Sagili RR, Pankiw T, Metz BN (2011). Division of labor associated with brood rearing in the honey bee: how does it translate to colony fitness?. PLoS One.

[CR78] Sakagami SF (1958). The false-queen: fourth adjustive response in dequeened honeybee colonies. Behaviour.

[CR79] Seehuus S-C, Norberg K, Krekling T, Fondrk K, Amdam GV (2007) Immunogold localization of vitellogenin in the ovaries, hypopharyngeal glands and head fat bodies of honeybee workers, *Apis mellifera*. J Insect Sci 7:1–14. doi:10.1673/031.007.5201 10.1673/031.007.5201 PMC2999438 20337562

[CR80] Seeley TD (1982). Adaptive significance of the age polyethism schedule in honeybee colonies. Behav Ecol Sociobiol.

[CR81] Seeley TD (1995). The wisdom of the hive : the social physiology of honey bee colonies.

[CR82] Seeley TD, Kolmes SA (1991). Age polyethism for hive duties in honey bees—illusion or reality?. Ethology.

[CR83] Slessor KN, Winston ML, Le Conte Y (2005). Pheromone communication in the honeybee (*Apis mellifera* L.). J Chem Ecol.

[CR84] Snodgrass RE (1925). Anatomy and physiology of the honeybee.

[CR85] Tanaka ED, Hartfelder K (2004). The initial stages of oogenesis and their relation to differential fertility in the honey bee (*Apis mellifera*) castes. Arthropod Struct Dev.

[CR86] Traynor K (2014) Decoding brood pheromone: the releaser and primer effects of young and old larvae on honey bee (*Apis mellifera*) workers. Thesis, Arizona State University

[CR87] Tsuruda JM, Amdam GV, Page RE (2008). Sensory response system of social behavior tied to female reproductive traits. PLoS One.

[CR88] Wang Y, Kaftanoglu O, Siegel AJ, Page RE, Amdam GV (2010). Surgically increased ovarian mass in the honey bee confirms link between reproductive physiology and worker behavior. J Insect Physiol.

[CR89] Wang Y, Kaftanoglu O, Fondrk MK, Page RE, Jr. (2014) Nurse bee behaviour manipulates worker honeybee (*Apis mellifera* L.) reproductive development. Anim Behav 92:253–261. doi:10.1016/j.anbehav.2014.02.012

[CR90] Wegener J, Huang Z, Lorenz MW, Bienefeld K (2009). Regulation of hypopharyngeal gland activity and oogenesis in honey bee (*Apis mellifera*) workers. J Insect Physiol.

[CR91] Whitfield CW, Cziko AM, Robinson GE (2003). Gene expression profiles in the brain predict behavior in individual honey bees. Science.

[CR92] Willis L, Winston M, Slessor K (1990). Queen honey bee mandibular pheromone does not affect worker ovary development. Can Entomol.

[CR93] Wilson EO, Bossert WH (1963). Chemical communication among animals. Rec Prog Hormone Res.

[CR94] Winston ML, Slessor KN (1998). Honey bee primer pheromones and colony organization: gaps in our knowledge. Apidologie.

[CR95] Zheng H-Q, Dietemann V, Crewe RM, Hepburn R, Hu F-L, Yang M-X, Pirk CWW (2010). Pheromonal predisposition to social parasitism in the honeybee *Apis mellifera* capensis. Behav Ecol.

