# Supplementary figures and images for: Queen and young larval pheromones impact nursing and reproductive physiology of honey bee (Apis mellifera) workers
Source: Behav Ecol Sociobiol. 2014 Sep 25;68(12):2059–73. doi: 10.1007/s00265-014-1811-y (PMC4220115; doi:10.1007/s00265-014-1811-y)

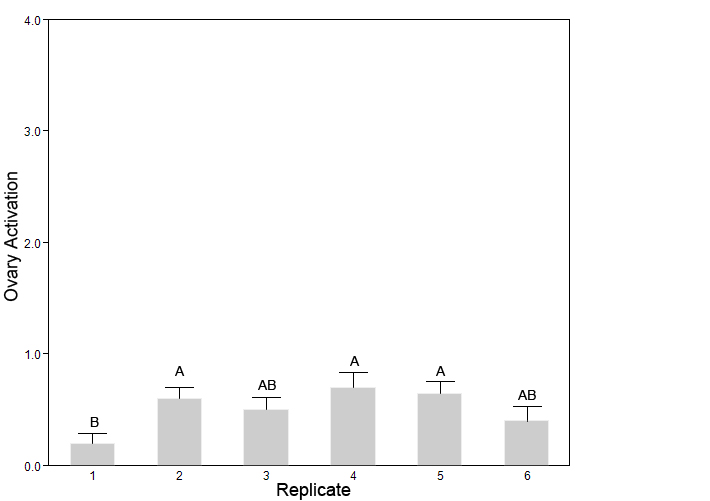

Supplement: Supplementary file 1 — Experiment 2: access to royal jelly vs. nurse bee mean (+S.E.) ovary activation by replicated. N = 180 bees, 60 per treatment, 30 per replicate, 10 bees per cage. Different letters indicate significant differences using LSD student t tests. (JPEG 60 kb) [file 265_2014_1811_Fig7_ESM.jpg]

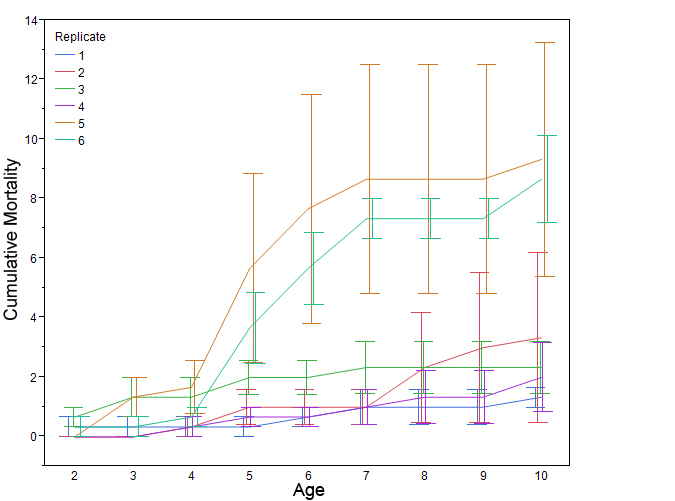

Supplement: Supplementary file 2 — Experiment 3: eβ dose cumulative mortality (+S.E.) per cage over 10 days. N = 180 bees, 60 per treatment, 30 per replicate, 10 bees per cage. (JPEG 136 kb) [file 265_2014_1811_Fig8_ESM.jpg]

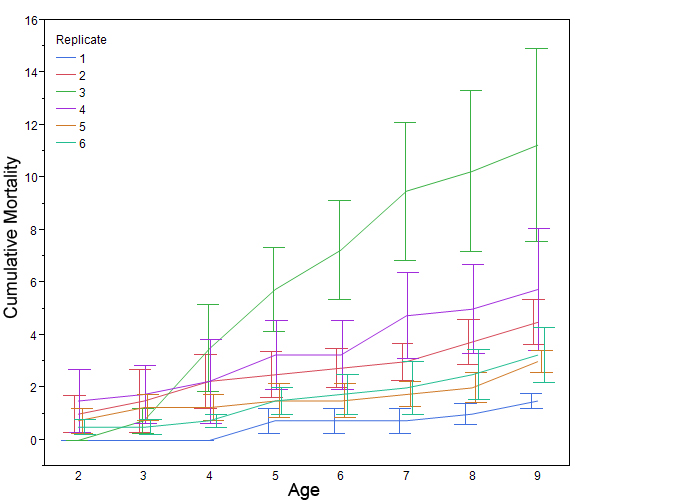

Supplement: Supplementary file 3 — Experiment 4: eβ & QMP cumulative mortality (+S.E.) per cage over 10 days. N = 240 bees, 60 per treatment, 30 per replicate, 10 bees per cage. (JPEG 137 kb) [file 265_2014_1811_Fig9_ESM.jpg]
